# The GroEL chaperonin: a protein machine with pistons driven by ATP binding and hydrolysis

**DOI:** 10.1098/rstb.2017.0179

**Published:** 2018-05-07

**Authors:** George H. Lorimer, Xue Fei, Xiang Ye

**Affiliations:** 1 Biophysics Graduate Program, University of Maryland, College Park, MD 20742, USA; 2 Center for Biological Structure and Organization, University of Maryland, College Park, MD 20742, USA; 3 Biochemistry Graduate Program, University of Maryland, College Park, MD 20742, USA; 4 Department of Chemistry and Biochemistry, University of Maryland, College Park, MD 20742, USA; 5 Institute for Physical Science and Technology, University of Maryland, College Park, MD 20742, USA

**Keywords:** allostery, helix–dipole, chaperonin

## Abstract

In response to the binding of ATP, the two heptameric rings of the GroEL chaperonin protein interact with one another in a negatively cooperative manner. Owing to the helix dipole, the positively charged nitrogen of glycine 88 at the N-terminus of helix D binds to oxygen atoms on the *β* and *γ* phosphorus atoms of ATP. In apo-GroEL, the nucleotide-binding sites of different rings are connected to one another by the interaction of the ɛ-amino group of lysine 105 of one helix D across the twofold axis with the negatively charged carbonyl oxygen atom of alanine 109 at the C-terminus of the other helix D. Upon binding ATP, the K105–A109 salt bridge breaks and both helices move apart by approximately 3.5 Å en bloc toward the ATP. Upon hydrolysis of ATP, the helices return to their original position. The helices thus behave as pistons, their movement being driven by the binding and hydrolysis of ATP.

This article is part of a discussion meeting issue ‘Allostery and molecular machines’.

## 1. Introduction

One of the most intriguing aspects of the operation of the GroEL chaperonin nano-machine concerns the manner in which the two heptameric rings communicate with one another. This is manifest by two related allosteric phenomena, the negatively cooperative binding of ATP to the two rings [1] and the breakage of symmetry, i.e. the conversion of the symmetric, football-shaped GroEL–GroES_2_ complex to the asymmetric, bullet-shaped GroEL–GroES_1_ complex [2,3] that depends upon the stochastic hydrolysis of ATP [4] and the development of nucleotide asymmetry between the rings.

The inter-ring interface across which the negative cooperativity must be communicated has several types of interaction. Two inter-ring salt bridges may be important in stabilizing the interface and/or in permitting allosteric communication; E461–R452 at the so-called right site [5–7] may serve to stabilize the interface, while the salt bridge involving helix D at the left site may communicate signals between the rings.

**Figure 1.**
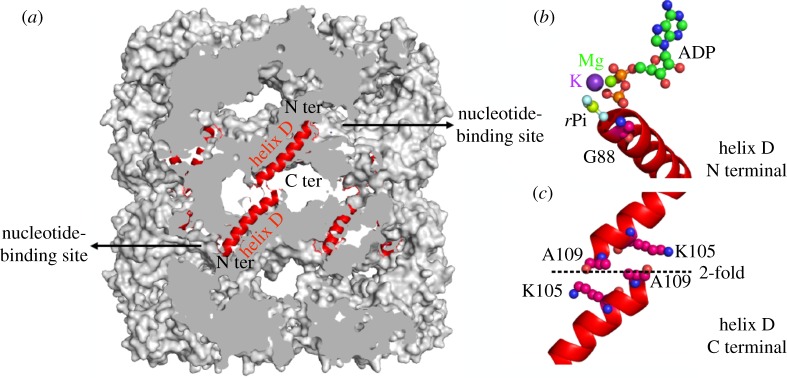
(*a*) A sagittal section through apo-GroEL revealing how two nucleotide-binding sites of the different rings communicate allosterically with one another via the helix dipoles of helix D. (*b*) Owing to the helix dipole, the Gly88 main chain nitrogen atom at the N-terminus of helix D possesses a partial positive charge, suitable for interaction with the βγ-phosphates of ATP. (*c*) Owing to the helix dipole, the carbonyl oxygen of Ala109 at the C-terminus of helix D possesses a partial negative charge that forms an electrostatic interaction across the twofold axis with the ɛ-amino group of Lys105.

The 21-residue helix D (Gly88–Ala109) extends from the nucleotide-binding site to the twofold axis of symmetry between the rings (figure 1) [6,7]. This helix forms a dipole, positively charged on the N-terminal N of Gly88, negatively charged on the C-terminal carbonyl oxygen of Ala109. In apo-GroEL a salt bridge forms, extending from the ɛ amino group of Lys 105 of one ring across the twofold axis of symmetry to the carbonyl oxygen of Ala109 of the other ring [8]. Although this is not a strong salt bridge by standard criteria [9,10], in apo-GroEL it is repeated 14 times. The negative cooperativity with respect to ATP binding to the different rings is transmitted from one ring to the other via these inter-ring Lys105–Ala109 salt bridges. The importance of these salt bridges was demonstrated with the mutant Lys105Ala, which displays no negative cooperativity between the subunits of different rings but unaltered positive cooperativity between the subunits of the same ring [8].

There are many structures of GroEL in the Protein Data Bank, both wild-type and mutant, with resolutions generally ranging from 2–8 to 3.8 Å (table 1) [3,6,7,11–15]. These GroEL_14_ structures can be classified into three groups: structures containing no GroES_7_; asymmetric structures, colloquially known as ‘bullets’, containing a single GroES_7_; and symmetric structures (‘footballs’) containing two molecules of GroES_7_. These structures are further defined by the presence or absence in one or both GroEL rings of ADP, ATP or molecules such as ADP.BeF_X_ thought to mimic ATP [16]. These structures are germane to the physiological function of GroEL that, depending on the absence or presence of unfolded substrate protein, operates via an asymmetric or symmetric cycle, respectively [17,18].

**Table 1.** Inter-ring distances between main and side chain atoms of helix D in various high-resolution structures of GroEL.

entry		PDB code	method	resolution	distance (Å) G88(N)–G88(N)	distance (Å) A106(C*α*)– A106(C*α*)	distance (Å) K105(*ɛ*N)–A109(O)
1	apo-wild-type	1XCK	x-ray	2.92	63.42 ± 0.12 *n* = 7	9.10 ± 0.14 *n* = 7	5.45 ± 0.27 *n* = 14
2	apo-K105A	4WSC	x-ray	3.04	63.42 ± 0.21 *n* = 7	8.80 ± 0.21 *n* = 7	not measurable
3	apo-D83A/R197A	4WGL	x-ray	3.13	63.34 ± 0.26 *n* = 7	8.77 ± 0.13 *n* = 7	4.47 ± 0.45 *n* = 14
4	ADP_14_ D83A/R197A	4KI8	x-ray	2.72	62.46 ± 0.42 *n* = 7	9.06 ± 0.10 *n* = 7	4.13 ± 0.52 *n* = 14
5	apo-wild-type	5WOS	cryo-EM	3.5	63.23 ± 0.30 *n* = 3	8.71 ± 0.72 *n* = 3	4.90 ± 0.10 *n* = 3
6	ATP_14_ wild-type	4AB2–4AAU	cryo-EM	8–9	66.42 ± 1.33 *n* = 6	13.10 ± 1.50 *n* = 6	
7	‘bullet’ ADP_7 (*cis*)_	1AON	x-ray	3.0	63.31 ± 0.04 *n* = 7	9.73 ± 0.04 *n* = 7	5.84 ± 0.05 (*cis*) 10.02 ± 0.10 (*trans*)
8	‘football’ (ADP-BeF_3_)_14_	4PKN	x-ray	3.60	67.23 ± 0.17 *n* = 7	12.67 ± 0.07 *n* = 7	7.98 ± 1.17 *n* = 14
9	‘football’ (ADP-BeFx)_14_	4PKO	x-ray	3.84	66.74 ± 0.28 *n* = 7	12.38 ± 0.02 *n* = 7	7.88 ± 1.38 *n* = 14
10	‘football’ D52A/D358A (ATP)_14_	3WVL	x-ray	3.79	66.55 ± 0.23 *n* = 7	12.36 ± 0.08 *n* = 7	9.08 ± 1.21 *n* = 14

Here we explore the role of the dipole associated with helix D in facilitating the allosteric communication between the two nucleotide-binding sites. By measuring distances between atoms within each helix D across the twofold axis in these structures we conclude that helix D undergoes en bloc, piston-like movements in response to the binding and hydrolysis of ATP.

## 2. Results and discussion

During the course of a chaperonin cycle GroEL undergoes some quite spectacular domain movements. Although these are initiated by binding of ATP to the equatorial domain, the major domain movements occur in the intermediate and apical domains. By comparison, movements within the equatorial domain are much less obvious. Nevertheless, the origin of the negative cooperativity between the rings is surely based, at least in part, upon conformational changes in the equatorial domain, however subtle. Disruption of the inter-ring salt bridge between the ɛ-amino group of lysine 105 of one ring and the carbonyl oxygen of alanine 109 of the opposite ring brings about the loss of negative cooperativity in the mutant K105A [8]. A comparison of the structures of apo-GroEL^WT^ (PDB 1XCK) and apo-GroEL^K105A^ (PDB 4WSC) showed that, except for the absence of electron density associated with the lysyl side chain, the two structures are identical. Accordingly we undertook a much closer inspection of the structure of helix D in various conformational states (table 1). We first measured the distance from the main chain N of glycine 88 of helix D in one ring to the same atom in helix D of the opposite ring in four crystal structures and one cryo-EM structure (entries 1–5 of table 1). The mean distance for all five structures was 63.17 ± 0.36 Å (*n* = 7) (table 2). Similarly, the mean inter-ring distance between the ɛ amino group of lysine 105 of one ring and the carbonyl oxygen of alanine 109 in the opposite ring was 4.74 ± 0.49 Å (*n* = 14). We next measured the main chain Gly88(N)–Gly88(N) distances in the six cryo-EM structures (at 8–9 Å resolution) of GroEL–ATP_14_ (entry 6 of table 1). The mean distance was 66.42 ± 1.33 Å. We chose not to measure the inter-ring Lys105(*ɛ*N) to Ala109(O) distances because at this resolution the positions of the side chains are not known. From these measurements it appeared that Gly88(N) was moving apart from its counterpart in the opposite ring by approximately 3 Å. To confirm this result at higher resolution we measured these distances in the symmetric ‘football’ structures containing either ATP or the ATP mimic (ADP-BeFx) in the nucleotide-binding site (entries 8–10 of table 1). The mean Gly88(N)–Gly88(N) distance was 66.42 ± 1.33 Å (table 2), while the mean inter-ring Lys105(*ɛ*N)–Ala109(O) distance was 8.31 ± 0.54 Å. Several conclusions may be drawn. First, upon binding ATP the inter-ring Lys105(*ɛ*N)–Ala109(O) salt bridge connecting the two helices breaks. Second, upon binding ATP the two helix Ds move apart by some 3.57 Å (table 2), as do the components of the inter-ring Lys105(*ɛ*N)–Ala109(O) salt bridge. Note also that it is not the breakage of the inter-ring salt bridge that causes the helices to move apart, because the Gly88(N)–Gly88(N) distance in the mutant GroEL^K105A^ remains unaltered (table 1, entry 2). Nor does destabilizing the taut allosteric state by mutation of the two inter-domain Asp83–Lys327 and Arg197–Glu386 salt bridges cause the Gly88(N)–Gly88(N) distance to increase (entries 3 and 4, table 1).

**Table 2.** Mean inter-ring distances between main and side chain atoms of helix D in the absence of ATP (entries 1–5 of table 1) or presence of ATP (or ATP mimic ADP.BeFx) (entries 6, 8–10 of table 1).

	mean distance (Å)
Gly88(N)–Gly88(N)	
entry # 1–5	63.17 ± 0.36
entry # 8–10	66.74 ± 0.31
differential distance	3.57
Ala106(C*α*)–Ala106(C*α*)	
entry # 1–5	8.89 ± 0.16
entry # 8–10	12.47 ± 0.14
differential distance	3.58
Lys105(*ɛ*N)–Ala109(O)	
entry # 1–5	4.74 ± 0.49
entry # 8–10	8.31 ± 0.54
differential distance	3.57

There are several reasons why the two helix Ds move apart upon occupancy of the nucleotide-binding site by ATP. Perhaps the simplest of these might be if the entire 21-residue helix D moved en bloc away from its counterpart in the other ring. If this is the case then the distance differential should be independent of the position on the helix where the distance measurements are made. Accordingly, we repeated the inter-ring distance measurements employing C*α* of Ala106. In the absence of ATP, the mean Ala106(C*α*)– Ala106(C*α*)-distance was 8.89 ± 0.16 Å, whereas in the presence of ATP or ADP.BeFx this distance was 12.47 ± 0.14 Å (table 2), the same differential of 3.57 Å as observed before. We conclude that upon binding ATP, the two helices D move away from one another en bloc in the manner of pistons, perhaps as a consequence of the positively charged main chain N of Gly88 being attracted to the negatively charged oxygen atoms on the *γ* phosphorus of ATP.

If the two helices D move away from one another upon binding ATP, how do they behave upon hydrolysis of ATP? To address this question we examined the asymmetric ‘bullet’ complex 1AON (entry 7, table 1). This contains ADP in the *cis* ring, but is ligand-free in the *trans* ring. It thus corresponds to the acceptor state of the chaperonin ATPase cycle [16]. The Gly88(N)–Gly88(N) distance (entry 7, table 1) of this asymmetric complex is 63.31 ± 0.04 Å, within experimental error of the distance determined for apo-GroEL (entries 1–3, 5) and for the ADP-saturated, allosterically compromised, double mutant GroEL^D83A/R197A^ (entry 4, table 1). At 9.73 ± 0.04 Å, the Ala106(C*α*)–Ala106(C*α*) of the asymmetric complex is slightly longer than the corresponding distances measured for the apo-GroELs. However, the inter-ring salt bridge extending from the *cis* K105(*ɛ*N) to the *trans* A109(O) was measured at 5.84 ± 0.05 Å, close enough to form seven weak salt bridges. On the other hand, at 10.02 ± 0.10 Å the distance from *trans* K105(*ɛ*N) to the *cis* A109(O) precludes the existence of a salt bridge. We conclude that, upon ATP hydrolysis, the helix D undergoes a reverse piston-like en bloc movement, returning to its original position.

In the presence of substrate protein, the chaperonin system operates via the symmetric cycle [17] in which the predominant form is the symmetric GroEL–GroES_2_ complex [2,3]. Continued cycling requires that this symmetric complex undergoes breakage of symmetry, losing one or other GroES so as to revert to the asymmetric acceptor-state, the GroEL–GroES_1_ complex [2,3]. This breakage of GroES symmetry requires the hydrolysis of not just one but several of the 14 ATP molecules originally present in the symmetric complex [2,3]. Failure to hydrolyze ATP, as in the ATPase-deficient, double mutant GroEL^D52A/D358A^, stabilizes the symmetric complex and enables its crystallization (entry 10, table 1) [15].

The breakage of several of the βγ phosphorus anhydride bonds of ATP alone appears insufficient to permit the reverse movement of helix D. Instead the progressive dissociation of the product Pi is additionally necessary. But dissociation of Pi and the movement of helix D are not strictly coupled. Dissociation of Pi must precede the binding of BeF_3_ to create the ATP-like ADP–BeF_3_ complex that effectively locks the helix D pistons in their extended conformation (entries 7 and 8, table 1).

What role might be played by the other inter-ring salt bridge, that between E461 of one ring and R452 of the other ring? To answer this, we measured the distance between the carboxyl oxygen (OE2) of E461 and the guanidine nitrogen (NH2) of R452 in the apo-protein (wild-type and the mutant K105A) in the asymmetric GroEL–GroES_1_ complex and in the symmetric GroEL–GroES_2_ complex (table 3). In both apo-structures and in the symmetric complex, this distance remained constant at about 3.5 Å, a strong salt bridge by standard criteria [9,10]. Small distance changes were detected in the asymmetric complex; the *cis*-ring E461(OE2)–*trans*-ring R452(NH2) distance decreases to 2.60 Å while the reciprocal distance lengthens to 6.24 Å. The latter may form seven weak longer-range ion pairs [10]. Since there are 14 such salt bridges it seems probable that they make a considerable contribution to the stability of inter-ring interface that persists throughout the chaperonin cycle, regardless of the allosterically driven movements of helix D.

**Table 3.** Inter-atomic, inter-ring distances (Å) between E461(OE2) and R452(NH2) of GroEL in three structural states: wild-type and the mutant K105A that lacks negative cooperativity (entries 1 and 2), the asymmetric GroEL–GroES1 ‘bullet’ complex (entry 3) and the symmetric GroEL–GroES2 complex (entry 4).

Entry		PDB Code	method	resolution	distance (Å) E461(OE2)–R452(NH2)
1	Apo-wild-type	1XCK	x-ray	2.92	3.35 ± 0.21 *n* = 14
2	Apo-K105A	4WSC	x-ray	3.04	3.35 ± 0.35 *n* = 14
3	‘Bullet’ ADP_7_ (*cis*)	1AON	x-ray	3.0	2.60 ± 0.10 (*cis*–*trans*) *n* = 7 6.24 ± 0.12 (*trans*–*cis*)
4	‘Football’ (ADP-BeFx)_14_	4PKO	x-ray	3.84	3.84 ± 0.63 *n* = 14

The piston-like movement of helix D in response to ATP binding and hydrolysis partly explains the allostery between the nucleotide binding sites in the equatorial domains of the GroEL rings. However, this movement is only part of a remarkable long-ranged, inter-domain allosteric network that enables GroEL to communicate within and between the chaperonin rings. The manner in which, for example, the binding of ATP to the nucleotide-binding sites in the equatorial domain of one ring is sensed some 95 Å distant in the apical domain of the other ring, leading to the dissociation of GroES, remains an unsolved mystery.

## Data accessibility

The data used was obtained from the Protein Data Base.
